# Editorial: Bariatric surgery, nutritional aspects and beyond

**DOI:** 10.3389/fnut.2023.1214952

**Published:** 2023-07-07

**Authors:** Edda Cava, Christina N. Boyle, Sofie Ahlin, Esmeralda Capristo

**Affiliations:** ^1^Dietetics and Clinical Nutrition, San Camillo Forlanini Hospital, Rome, Italy; ^2^Institute of Veterinary Physiology, Vetsuisse Faculty, University of Zurich, Zurich, Switzerland; ^3^Department of Molecular and Clinical Medicine, Institute of Medicine, Sahlgrenska Academy, University of Gothenburg, Gothenburg, Sweden; ^4^Department of Medical and Surgical Sciences, Fondazione Policlinico Universitario A. Gemelli IRCSS, Rome, Italy; ^5^Department of Translational Medicine and Surgery, Catholic University of the Sacred Heart, Rome, Italy

**Keywords:** gastric by-pass, nutritional deficiencies, obesity treatment, sleeve gastrectomy, weight regain

Obesity represents a global epidemic with significant human and financial impacts. Since 1975, its prevalence has almost tripled worldwide, accounting for at least 2.8 million deaths/year (1, 2). This is mainly attributable to the associated complications, such as type 2 diabetes, obstructive sleep apnoea, cardiovascular diseases, non-alcoholic fatty liver disease, cancer, osteoarthritis, and chronic kidney disease (3).

The current recommendation states that the resolution of obesity complications occurs with weight loss amounting to 5%−20% of total body weight, overcoming the 5% threshold previously indicated as a goal treatment (4).

Lifestyle interventions, including dietary modification and increased physical activity levels coupled with psychological support, represent the cornerstone of obesity treatment but are associated with poor results in the long term (5). In general, weight regain is common when lifestyle interventions only are implemented, and it is expected that ~80% of the weight that was lost is regained over the next 5 years (6). The development and use of new and potent drugs will revolutionize the medical treatment of obesity, even in association with less invasive endoscopic/bariatric procedures (7–9).

Currently, bariatric surgery (BS) is the most effective and sustained weight loss procedure, being able to induce long-term weight reduction of up to 30%, with consequent substantial amelioration or even long-term remission of type 2 diabetes and other obesity-related complications (10, 11).

A recently published first randomized trial compared the effects of BS with lifestyle modification *plus* improved medical care in people with histologically proven non-alcoholic-steatohepatitis (NASH), confirming the superiority of BS in treating obesity complications (11).

Complex mechanisms are involved in the early and late weight loss after BS, such as altered hormonal pathways, bile acid signaling, and changes in microbiota composition and its metabolites, all of which could play a role in the multiple improvements seen in patients after BS (10).

A growing body of literature is available regarding the two most common bariatric procedures performed, namely, laparoscopic Roux-en-Y gastric bypass (RYGB) and sleeve gastrectomy (SG) (6, 9).

The safety of BS has markedly improved over the past two decades, largely due to the adoption of the laparoscopic surgical approach, with a 10-fold decrease in surgical mortality rates compared to equivalent open operations (12). In addition, perioperative mortality rates from BS are less than those from laparoscopic cholecystectomy or appendectomy. Furthermore, the perioperative complication rate for laparoscopic BS was less than that reported for laparoscopic hysterectomy, cholecystectomy, or appendectomy (12).

On the other hand, the most common long-term complications of BS include nutritional deficiencies and their consequences, i.e. iron, calcium, vitamin, and microelement deficiency with increased risk of anemia, osteoporosis, fractures, fatigue, and alopecia (13).

Thus, the severity of nutritional impairment depends on both the type of surgery and patient compliance with follow-up and medical treatment.

As obesity is a chronic and progressive disease, weight loss response to surgery will vary individually and a proportion of patients experience insufficient weight loss or clinically significant weight regain after BS. The mechanisms at the basis of weight regain after BS may involve genetics, dysregulated/maladaptive eating behaviors, inadequate choice of operation, psychological factors, etc. (14). Estimating the prevalence of weight regain after BS is limited by a lack of consensus on its definition and a comprehensive evaluation by a multidisciplinary team is highly needed in these patients.

To shed some light on macronutrient and bile acid malabsorption after BS procedures, Evenepoel et al. compared RYGB and SG to controls using isotope technology and also investigated the impact of surgery on colonic protein fermentation. They showed how macronutrient malabsorption is limited and, although different between the two procedures, it does not affect the nutritional status of patients in both cases. In addition, the higher protein fermentation and slower bowel transit occurring after both BS procedures could affect colonic health, therefore suggesting that this field of research deserves more interest and attention.

The studies included in this Research Topic suggest that BS-related changes in colonic microbial metabolites might drive colorectal carcinogenesis due to altered dietary intake, macronutrient malabsorption, altered transit, and persistent low microbial diversity.

The research by Ismaeil et al. studied macronutrient intake in RYGB rats. In their study, ingestive adaptation and learning occurred over time after surgery, independently from early postoperative food intervention. They provided high-fat food to RYGB and control rats and found that it did not accentuate fat avoidance and did not lead to superior weight loss in the long term in RYGB-treated rats, showing how food preferences undergo progressive changes after BS.

Beyond the metabolic aspect, a prospective cohort study by Goldenshluger et al. investigated the short-term changes in mental, physical, and social factors associated with percentage excess-weight loss (%EWL) occurring after BS in adolescents. Using self-reported questionnaires, they found how a meaningful (~30%) decrease in BMI, not related to %EWL, induced multiple improvements in short-term physical, mental, and social factors and, remarkably, patients experienced benefits in mental health after undergoing BS when they had previous feelings of social rejection. Therefore, BS may have a positive impact on weight-related stigma, improving patients' quality of life in adolescents and adults.

Eventually, the future of personalized medical approaches through digital innovations could include the application of artificial intelligence systems analyzing multi-dimensional data to support patients in improving health and quality of life. Schönenberger et al. provided an example of the impact of artificial intelligence systems in patients with post-bariatric hypoglycemia after using automated food analysis connected to continuous glucose measuring and other health-related data to improve glucose control for patients who underwent BS.

In conclusion, BS has many benefits beyond weight loss, and optimizing the post-surgery follow-up is mandatory to maximize metabolic benefits and reduce the risks of complications and nutrient deficiencies.

This Research Topic on BS provides a brief update in clinical nutrition on the metabolic, psychological, and also digital aspects involved in the surgical treatment of severe obesity, and aims to provide useful information to improve the cure of patients undergoing bariatric procedures and to improve the risk-to-benefit ratio of BS.

## Author contributions

ECav, CNB, SA, and ECap contribute to the writing of the editorial. All authors contributed to the article and approved the submitted version.
